# Deciphering the Overlapping Immune Mechanism Between Depression and Breast Cancer

**DOI:** 10.3390/ijms26115229

**Published:** 2025-05-29

**Authors:** Yiming Ma, Yu Ming, Zhiyong Hou, Yanan Yu, Jun Liu, Zhong Wang

**Affiliations:** Institute of Basic Research in Clinical Medicine, China Academy of Chinese Medical Sciences, Beijing 100700, China; mym6650362@163.com (Y.M.); mingyu961221@163.com (Y.M.); houzhiyong2018@163.com (Z.H.); yuyanan@mail.cintcm.ac.cn (Y.Y.)

**Keywords:** depression, breast cancer, bioinformatics, mechanisms, immune infiltration

## Abstract

Depression and breast cancer (BC) demonstrate significant clinical comorbidity, yet their shared molecular mechanisms remain unclear, particularly regarding immune pathway regulation. This study systematically analyzed Depression-associated gene expression profiles (Gene Expression Omnibus (GEO) database) and BC transcriptomic data (The Cancer Genome Atlas (TCGA) database), identifying overlapping differentially expressed genes (DEGs). Functional enrichment (Gene Ontology (GO)/Kyoto Encyclopedia of Genes and Genomes (KEGG)) and protein–protein interaction (PPI) network analyses (STRING/Cytoscape) were employed to elucidate biological processes, followed by least absolute shrinkage and selection operator (LASSO) regression and receiver operating characteristic (ROC) curve validation to prioritize key genes. Immune infiltration patterns were assessed via the xCell algorithm, with Spearman correlation linking genes to immune subsets, and single-gene Gene Set Enrichment Analysis (GSEA) evaluating pathway activity. In total, 93 overlapping genes were identified, with predominant involvement in immune-related pathways being revealed by functional enrichment analysis. *BHLHE41*, *EpCAM*, and *GSTM2* were prioritized as mechanism-associated genes through integrated LASSO regression and ROC analyses. Significant correlations were observed between these genes and specific immune cell populations. GSEA further linked these genes to immune response pathways, suggesting their regulatory roles. These findings highlight immune dysregulation as a shared mechanism underlying Depression-BC comorbidity, providing a foundation for developing early diagnostic strategies and therapeutic strategies targeting both conditions.

## 1. Introduction

Depression, a prevalent neuropsychiatric disorder characterized by persistent low mood and anhedonia, affects over 350 million individuals globally, representing approximately 4.4% of the world’s population [1]. Breast cancer (BC), a heterogeneous malignant neoplasm originating from abnormal proliferation of mammary gland cells, has emerged as the most prevalent cancer diagnosis and leading cause of cancer-related mortality worldwide since 2020 [2]. This complex disease develops through synergistic interactions between genetic predisposition and environmental determinants [3]. A systematic review revealed that the global prevalence of Depression among BC patients reached 30.2% [4]. Epidemiological studies have established Depression as a potential causal factor in BC development, demonstrating a significantly elevated risk of BC incidence among Depression patients [5,6]. Furthermore, longitudinal cohort studies have revealed that BC patients with comorbid Depression experience poorer clinical outcomes compared to their non-depressed counterparts [7]. These compelling associations underscore the critical need to elucidate the molecular mechanisms underlying the Depression-BC relationship.

The genetic architectures of Depression and BC demonstrate both divergence and convergence. Large-scale genome-wide association studies (GWAS) have identified nearly 200 common risk loci for depression, though their effect sizes vary substantially depending on phenotypic definitions. Rare protein-truncating variants (PTVs) and disruptive missense mutations in loss-of-function intolerant genes (pLI ≥ 0.9) confer significant disease risk. While polygenic risk scores (PRS) and rare variants exhibit additive effects, PRS accounts for a greater proportion of heritability [8]. A multi-ancestry GWAS identified 53 novel Depression risk loci, with fine-mapping and multi-omics integration pinpointing 43 high-confidence genes including *DRD2* (dopamine receptor D2) and mitochondrial gene *NDUFAF3*, revealing substantial ancestral heterogeneity in genetic architecture [9]. In a meta-analysis of 135,458 Depression cases and 344,901 controls, researchers identified 44 independent risk loci encompassing genes involved in metabolic regulation (*OLFM4*, *NEGR1*), neuronal splicing (*RBFOX1*), and synaptic function (*LRFNS*). Immune-related signals emerged in the major histocompatibility complex (MHC) region. Strong associations in evolutionarily conserved regions (20.9-fold enrichment, *p* = 1.4 × 10^−15^) and splicing regulatory pathways highlight developmental and post-transcriptional mechanisms in Depression pathogenesis [10]. Through multi-omics Mendelian randomization integrating GWAS, eQTL, and mQTL data, Zhang et al. identified *ATG10* and *RCCD1* as core BC regulatory genes. *ATG10* demonstrated risk reduction via autophagy-related mechanisms (OR = 0.89–0.95, *p* < 1 × 10^−8^) mediated by methylation site cg17942617, while *RCCD1* methylation (cg01710897/cg04851675) correlated with improved survival (*p* = 0.010) [11]. Cross-tissue splicing quantitative trait locus analysis revealed 88 novel susceptibility genes through splice variants, including 17 distal to GWAS loci and 110 detectable only via intronic splicing alterations. Aberrant RNA splicing events (e.g., AZGP1-GJC3 fusion) influence BC pathogenesis through estrogen signaling and immune evasion pathways [12]. Notably, genome-wide analyses reveal positive genetic correlation between Depression and BC (rg ≈ 0.08), with shared heritability observed for both ER+ and ER− subtypes. Key shared loci cluster in 6p22.1 (encompassing *ZSCAN12* and *ABT1*) and MHC regions (*HLA-S*, *FLOT1*), implicating immune–inflammatory pathways. Additional loci at 9q31.2 (*KLF4*) and rs56101042 (14q32.32 near *TRAF3*) suggest epigenetic and inflammatory mechanisms [13]. Mendelian randomization analyses further support a causal effect of Depression on BC risk (OR = 1.09–1.12), persisting after adjustment for smoking and alcohol consumption [6].

Previous studies have established a significant association between Depression and BC development, mediated through multiple pathophysiological mechanisms including chronic inflammation, oxidative stress, immune dysregulation, and hypothalamic–pituitary–adrenal (HPA) axis dysfunction [14]. Depression exhibits close associations with elevated systemic inflammatory cytokines including interleukin-6 (IL-6), IL-1β, tumor necrosis factor-α (TNF-α), and C-reactive protein (CRP) [15]. Peripheral pro-inflammatory cytokines can traverse the blood–brain barrier to activate neuroglial cells, triggering central neuroinflammation that disrupts synaptic plasticity and monoaminergic neurotransmission [16]. Persistent inflammatory signaling plays pivotal roles throughout cancer pathogenesis, where cytokine-mediated activation of epigenetic mechanisms (DNA, miRNA, and lncRNA regulation) in epithelial cells modulates oncogene and tumor suppressor expression [17]. Chronic stress induces excessive reactive oxygen species (ROS) accumulation, compromising DNA repair mechanisms and driving malignant transformation of mammary epithelial cells [17,18]. Concurrently, oxidative stress contributes to Depression pathogenesis through cerebral functional alterations, impaired neuronal plasticity, and volumetric reductions in the prefrontal cortex and hippocampus [19]. Genome-wide analyses demonstrate significant enrichment of immune-related pathways (e.g., IL-6 signaling and natural killer cell pathways) in patients with Depression [20]. Stress-induced behavioral changes are accompanied by microglial pruning of synaptic elements, potentially mediating neuroplasticity deficits. Both microglia and macrophages, as innate immune components, show distinct associations with stress vulnerability versus resilience [21]. Clinical evidence indicates that Depression compromises immune surveillance, increasing BC recurrence, metastasis risk, and mortality [22,23]. Immune checkpoint inhibitors (ICIs) demonstrate enhanced antitumor efficacy in early-stage triple-negative BC (TNBC) and PD-L1+ HR+/ERBB2- tumors [24]. The HPA axis dysfunction, hallmarking Depression pathophysiology, also exhibits oncogenic potential in BC development [25]. Cortisol dysregulation during HPA hyperactivity disrupts cellular growth signaling, with epidemiological studies revealing positive associations between flattened diurnal cortisol rhythms and BC risk [26,27,28,29,30]. Although emerging evidence has begun to delineate these mechanisms in Depression-BC comorbidity, the precise molecular pathways underlying Depression and BC remain largely elusive. Leveraging advanced bioinformatics approaches, we conducted comprehensive analyses to identify molecular signatures linking Depression and BC pathogenesis, with a focus on immune-inflammatory crosstalk, to lay the groundwork for novel strategies in prevention, early diagnosis, and targeted therapeutic interventions.

## 2. Results

### 2.1. Identification of Differentially Expressed Genes (DEGs) in Depression and Breast Cancer (BC)

**Figure 1:**
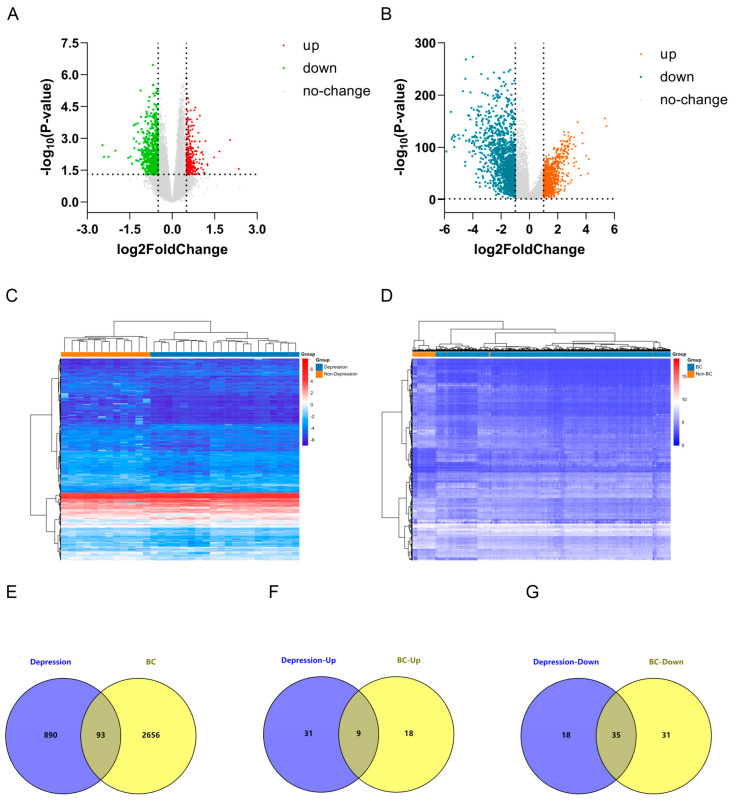
Depression and breast cancer (BC) differentially expressed genes (DEGs) analysis. (**A**) Volcano plot of DEGs based on Depression dataset GSE76826. (**B**) Volcano plot of BC DEGs based on The Cancer Genome Atlas (TCGA) dataset. (**C**) Heatmap of Depression DEGs analysis results based on GSE76826 dataset. (**D**) Heatmap of BC DEGs analysis results based on TCGA dataset. (**E**) Identification of DEGs in Depression and BC. (**F**) Identification of co-upregulated DEGs. (**G**) Identification of co-downregulated DEGs.

Differential expression analysis identified 983 DEGs in the Depression dataset (GSE76826), comprising 293 upregulated and 690 downregulated genes (Figure 1A). In the BC dataset (The Cancer Genome Atlas (TCGA) database), 2749 DEGs were detected, including 964 upregulated and 1785 downregulated genes (Figure 1B). Hierarchical clustering revealed distinct expression patterns for these DEGs in both conditions (Figure 1C,D). Comparative analysis identified 93 overlapping DEGs in Depression and BC, including 9 consistently upregulated and 35 consistently downregulated genes (Figure 1E–G), which may represent crucial molecular regulators in Depression and BC pathogenesis.

### 2.2. Results of Enrichment Analysis of Overlapping Genes

**Figure 2:**
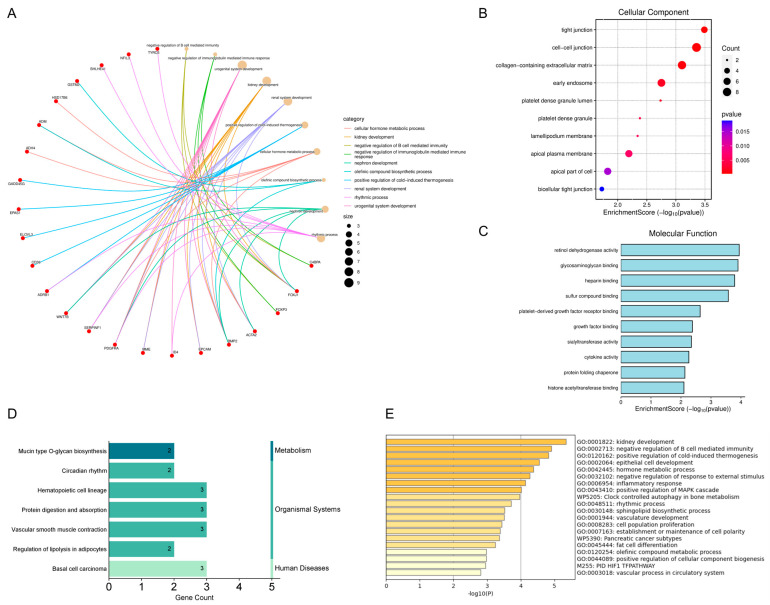
Pathway enrichment and functional enrichment of Depression and BC DEGs. (**A**) Significant enrichment of Gene Ontology (GO)-biological processes (BP). (**B**) Significant enrichment of GO-cellular components (CC). (**C**) Significant enrichment of GO-molecular functions (MF). (**D**) Significant enrichment of Kyoto Encyclopedia of Genes and Genomes (KEGG) pathway. (**E**) Enrichment analysis of 93 DEGs using Metascape online tool, https://metascape.org/, accessed on 3 March 2025.

**Figure 3:**
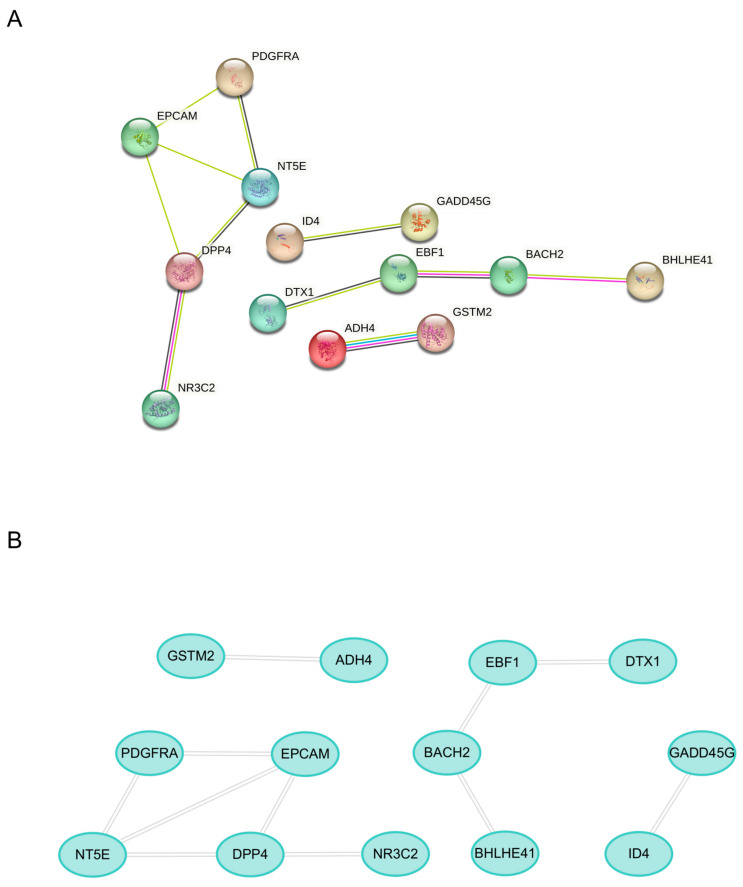
Screening of hub genes. (**A**) Protein–protein interaction (PPI) network of overlapping genes. Nodes represent genes; edges indicate interaction confidence (STRING score ≥ 0.4). (**B**) Network construction using Cytoscape software. The connected lines signify the potential existence of either a direct physical binding or a functional association between two proteins.

Gene Ontology (GO) and Kyoto Encyclopedia of Genes and Genomes (KEGG) enrichment analyses revealed that these genes were primarily associated with mucin-type O-glycan biosynthesis, negative regulation of B cell-mediated immunity, and negative regulation of immunoglobulin-mediated immune response (Figure 2A–D). Metascape-based analysis further corroborated the significant involvement of immune response pathways in Depression and BC pathogenesis [31] (Figure 2E). Through integrated analysis using the STRING database and Cytoscape, we identified 13 hub genes: *ADH4*, *GSTM2*, *BACH2*, *BHLHE41*, *EBF1*, *DPP4*, *NT5E*, *EpCAM*, *NR3C2*, *DTX1*, *PDGFRA*, *GADD45G*, and *ID4* (Figure 3). These hub genes may represent critical regulatory nodes in the molecular network underlying Depression and BC.

### 2.3. Machine Learning for Identification and Validation

**Figure 4:**
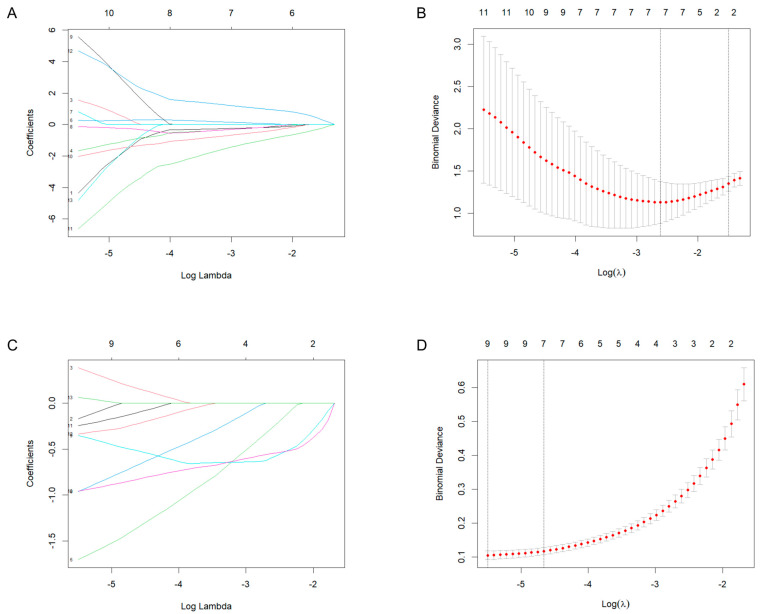
Identification of candidate genes by Least absolute shrinkage and selection operator (LASSO). (**A**,**B**) LASSO regression analysis of the Depression-GSE76826 dataset. (**C**,**D**) LASSO regression analysis of the BC-TCGA dataset. The left vertical dotted line (λ.min) indicates the model with the minimum cross-validation error. The right vertical dotted line (λ.1se) represents the most simplified model.

**Figure 5:**
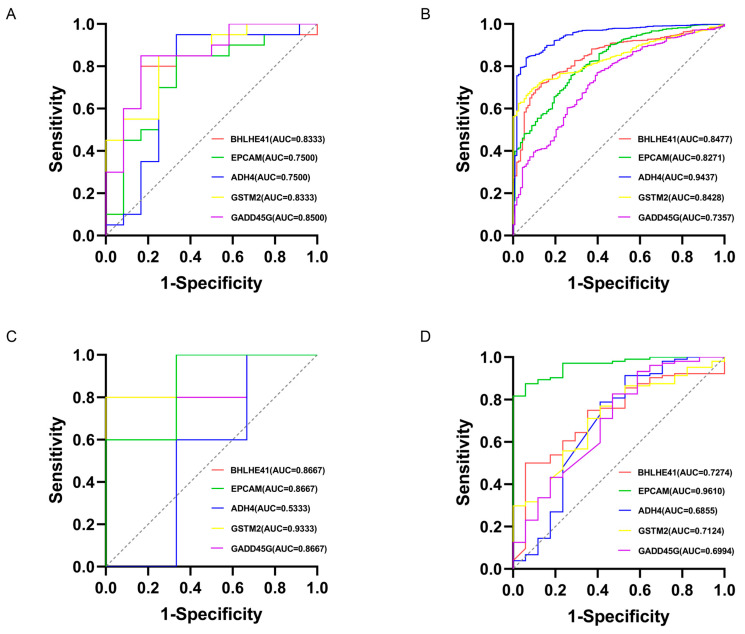
Results of receiver operating characteristic (ROC) analysis. (**A**) ROC curves of five candidate genes in the Depression-GSE76826 dataset. (**B**) ROC curves of 5 candidate genes in the BC-TCGA dataset. (**C**) ROC curves of 5 candidate genes in the Depression-GSE169459 validation set. (**D**) ROC curves of 5 candidate genes in the BC-GSE42568 validation set.

Least absolute shrinkage and selection operator (LASSO) regression identified five candidate genes (*BHLHE41*, *EPCAM*, *ADH4*, *GSTM2*, *GADD45G*) from the Depression and BC datasets (Figure 4). Receiver operating characteristic (ROC) analysis demonstrated robust diagnostic performance across multiple datasets. In the Depression-GSE76826 dataset, the area under the curve (AUC) values were *BHLHE41* (0.8333), *EPCAM* (0.7500), *ADH4* (0.7500), *GSTM2* (0.8333), and *GADD45G* (0.8500) (Figure 5A). The BC-TCGA dataset showed improved performance: *BHLHE41* (0.8477), *EPCAM* (0.8271), *ADH4* (0.9437), *GSTM2* (0.8428), and *GADD45G* (0.7357) (Figure 5B). Further validation was conducted using independent datasets. In the Depression-GSE169459 validation set, the AUC values were *BHLHE41* (0.8667), *EPCAM* (0.8667), *ADH4* (0.5333), *GSTM2* (0.9333), and *GADD45G* (0.8667) (Figure 5C). Validation in the BC-GSE42568 dataset confirmed consistent performance: *BHLHE41* (0.7274), *EPCAM* (0.9610), *ADH4* (0.6855), *GSTM2* (0.7124), and *GADD45G* (0.6994) (Figure 5D). Overall, *BHLHE41*, *EPCAM*, and *GSTM2* consistently suggested their potential as reliable mechanism-associated genes for Depression and BC, with AUC values exceeding 0.7 across both training and validation datasets.

### 2.4. Literature Validation

To validate the biological relevance of mechanism-associated genes, we conducted a comprehensive literature review using the PubMed database to investigate their reported associations with Depression and BC. The literature mining analysis revealed that *BHLHE41* and *GSTM2* have been previously implicated in both Depression and BC pathogenesis, while *EPCAM* has been primarily associated with BC. These findings are systematically summarized in Table 1, providing supporting evidence for the potential roles of these genes in Depression and BC.

### 2.5. Immune Cell Infiltration and Correlation Analysis

**Figure 6:**
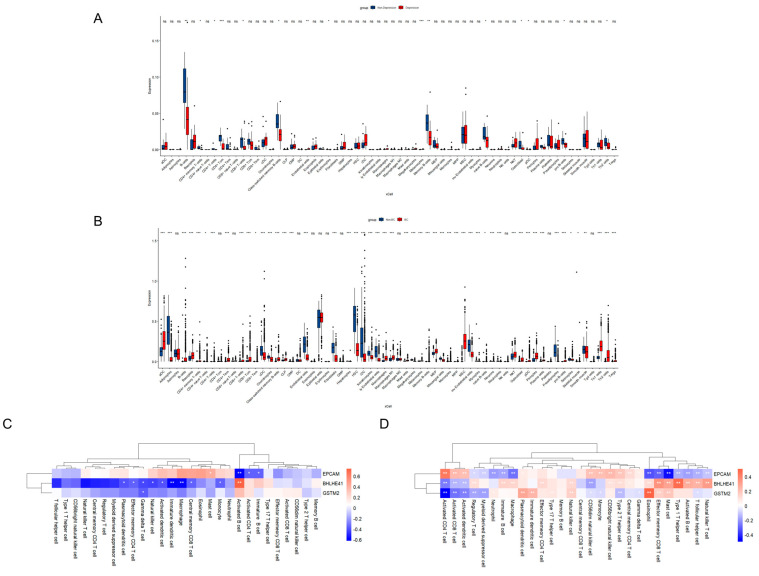
Correlation of mechanism-associated genes with immune cell infiltration in Depression and BC. (**A**) Boxplot of immune cell infiltration patterns in Depression-GSE76826 dataset. (**B**) Boxplot of immune cell infiltration patterns in BC-TCGA dataset. Blue color represents normal subjects and red color represents Depression/BC patients. (**C**) Heatmap of the correlation between mechanism-associated genes and immune cells in Depression-GSE76826 dataset. (**D**) Heatmap of the correlation between mechanism-associated genes and immune cells in BC-TCGA dataset. Red color indicates positive correlation and blue color indicates negative correlation. * *p* < 0.05; ** *p* < 0.01; *** *p* < 0.001; ns, not significant.

Given the critical role of immune function in Depression and BC pathogenesis revealed by enrichment analysis, we investigated immune response patterns in both conditions. Comparative analysis revealed contrasting immune cell profiles between Depression and BC relative to normal controls. Specifically, B-cell lineages (including naive B-cells, memory B-cells, and class-switched memory B-cells), CD8+ T-cells, endothelial cells, osteoblasts, and Th2 cells were significantly downregulated in Depression compared to healthy controls, while showing marked upregulation in BC samples (Figure 6A,B). These immune profiles suggest immunological mechanisms underlying Depression and BC pathogenesis.

While immune cell composition alterations represent one aspect of the pathogenic mechanisms, further investigation of mechanism-associated genes and immune cell interactions is crucial to understand their immunological implications. Correlation analysis revealed immune interaction patterns between Depression and BC datasets. In the Depression dataset, *BHLHE41* showed significant positive correlation with activated B cells (*p* < 0.01) but negative correlations with macrophages (*p* < 0.01), natural killer cells (*p* < 0.05), and effector memory CD4+ T cells (*p* < 0.05). *EpCAM* exhibited positive correlation with mast cells (*p* < 0.05) but negative correlations with activated CD4+ T cells (*p* < 0.05) and activated B cells (*p* < 0.01), patterns that were inversely observed in the BC dataset (*p* < 0.01) (Figure 6C,D). Additional analyses identified consistent negative correlations between *BHLHE41* and activated dendritic cells (for Depression: *p* < 0.05; for BC: *p* < 0.01), *EpCAM* and immature B cells (for Depression: *p* < 0.05; for BC: *p* < 0.01), and *GSTM2* and γδ T cells (*p* < 0.05) across both datasets (Figure 6C,D). These findings suggest that these genes may regulate autoimmune processes through modulation of immune cell expression profiles.

**Figure 7:**
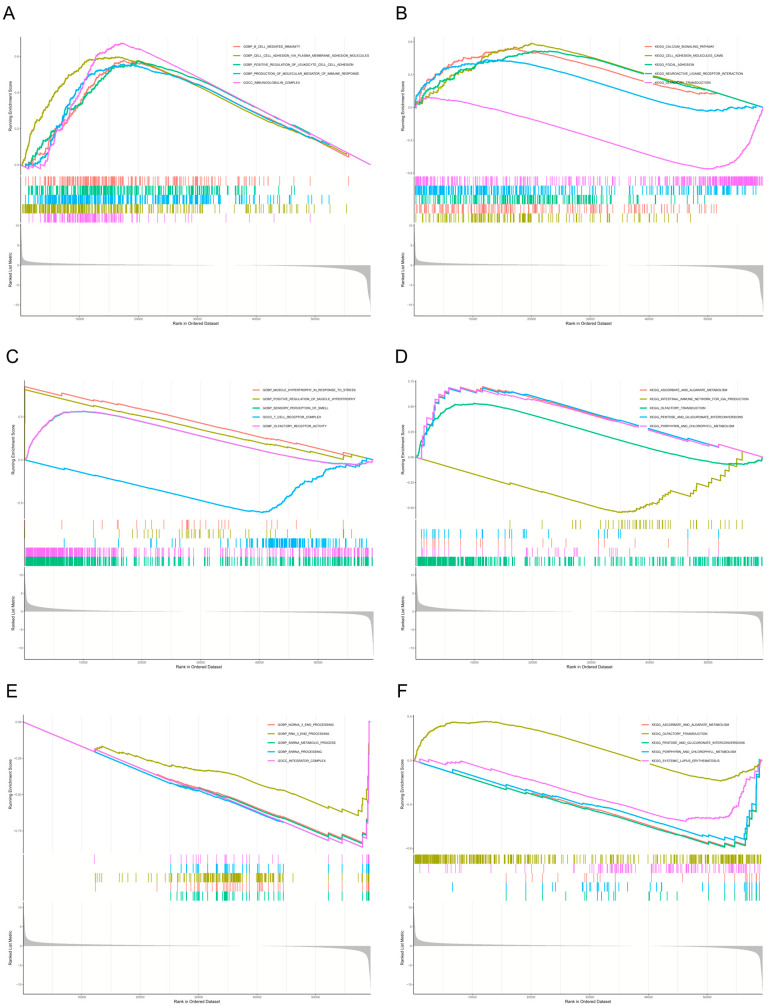
Results of single-gene Gene Set Enrichment Analysis (GSEA) analysis for mechanism-associated genes. (**A**,**B**) Results of single-gene GSEA analysis of *BHLHE41*. (**C**,**D**) Results of single-gene GSEA analysis of *EpCAM*. (**E**,**F**) Results of single-gene GSEA analysis of *GSTM2*.

Additionally, single-gene Gene Set Enrichment Analysis (GSEA) analysis revealed significant enrichment of *BHLHE41* in GO terms related to B cell-mediated immunity, cell–cell adhesion via plasma membrane adhesion molecules, positive regulation of leukocyte adhesion, production of immune response molecular mediators, and immunoglobulin complexes. KEGG analysis of *BHLHE41* indicated enrichment in pathways such as calcium signaling, cell adhesion molecules, focal adhesion, neuroactive ligand–receptor interactions, and olfactory transduction (Figure 7A,B). For *EpCAM*, GO analysis showed enrichment in stress-induced muscle hypertrophy, positive regulation of muscle hypertrophy, olfactory perception, T cell receptor complex, and olfactory receptor activity. KEGG analysis of *EpCAM* highlighted pathways including ascorbate and aldarate metabolism, intestinal immune network for IgA production, olfactory transduction, pentose and glucuronate interconversions, and porphyrin and chlorophyll metabolism (Figure 7C,D). As for *GSTM2*, GO analysis revealed enrichment in ncRNA 3′-end processing, RNA 3′-end processing, snRNA metabolic processes, snRNA processing, and integrator complex. KEGG analysis of *GSTM2* demonstrated enrichment in pathways such as ascorbate and aldarate metabolism, olfactory transduction, pentose and glucuronate interconversions, porphyrin and chlorophyll metabolism, and systemic lupus erythematosus (Figure 7E,F). These results suggest that these genes may play roles in immune processes involved in the pathogenesis of Depression and BC.

## 3. Discussion

By integrating datasets from GEO and TCGA databases, we employed differential expression profiling, protein–protein interaction network topology screening, LASSO regression modeling, and ROC curve validation to identify *BHLHE41*, *EpCAM*, and *GSTM2* as mechanism-associated genes connecting Depression with BC. Their central role was further confirmed through external dataset validation and cross-referencing with the existing literature evidence. Functional enrichment analysis combined with immune microenvironment profiling revealed the cross-disease activation of the MAPK signaling pathway, while demonstrating that these three genes participate in disease pathogenesis by regulating immune regulatory axes, thereby serving as molecular bridges for comorbidity development. The final mechanistic model positions these genes as regulatory hubs and the MAPK pathway as a dynamic signaling framework, deconstructing the profound interconnection between depression and BC at the molecular network level. This work not only provides a mechanism-driven theoretical foundation for understanding comorbidity in heterogeneous diseases but also opens new avenues for pathway-based interdisciplinary diagnostic and therapeutic strategies.

Basic Helix-Loop-Helix Family Member E41 (*BHLHE41*), a key member of the basic helix-loop-helix (bHLH) transcription factor family, plays multifunctional roles in cellular homeostasis by regulating cell cycle progression, circadian rhythms, stress responses, and oncogenic processes. Molecular characterization reveals *BHLHE41*’s dual role as both a downstream effector and modulator of p38 MAPK signaling, where its downregulation initiates a pathogenic cascade involving p38 MAPK-mediated microglial activation and subsequent neuroinflammatory responses through elevated pro-inflammatory cytokines (IL-6, IL-1β, TNF-α), while simultaneously impairing monoaminergic neurotransmission (notably 5-HT signaling) to disrupt emotional regulation [51,52,53,54]. Experimental evidence demonstrates that *BHLHE41* silencing induces robust activation of the JNK signaling pathway, triggering neuronal apoptosis and dendritic atrophy in limbic system structures, particularly the amygdala and hippocampus, which mechanistically contributes to depression-like pathophysiology [55]. Pharmacological inhibition of the MAPK/JNK signaling pathway rescued the impaired tumor cell invasiveness caused by *BHLHE41* knockdown, suggesting that *BHLHE41* primarily promotes MCF-7 cell invasion via activation of the MAPK/JNK axis [56]. Activation of the downstream p38 MAPK signaling in breast cancer cells upregulates epithelial–mesenchymal transition (EMT) markers and promotes tumor cell invasion [57]. Concurrently, *BHLHE41* modulates cancer progression through competitive binding at promoter regions of EMT master regulators (Snail, Slug, and Twist), thereby facilitating EMT-driven breast cancer cell invasion and metastasis [58,59]. These findings are complemented by recent work, collectively positioning *BHLHE41* as a critical node linking neuropsychiatric and oncological pathologies through shared signaling pathways.

Epithelial cell adhesion molecule (*EpCAM*), a single-pass type I transmembrane glycoprotein, serves as a critical regulator of intercellular adhesion and tissue homeostasis. In malignant transformation, *EpCAM* undergoes regulated intramembrane proteolysis, generating two functionally active fragments: the intracellular domain (EpICD) translocates to the nucleus where it forms a transcriptional complex with β-catenin, FHL2, and LEF1 to drive expression of genes associated with proliferation, stemness maintenance, and epithelial–mesenchymal plasticity, while the extracellular domain (EpEX) engages EGFR through its EGF-like motif to activate ERK/MAPK signaling, which simultaneously suppresses FOXO3a tumor suppressor activity through inhibition of nuclear translocation and stabilizes PD-L1 to establish an immunosuppressive microenvironment [60]. Pathologically elevated *EpCAM* expression orchestrates a coordinated oncogenic program involving HtrA2 downregulation-mediated apoptosis resistance, PD-L1 upregulation, Treg recruitment, and CD8+ T cell functional impairment, collectively facilitating immune evasion in breast carcinoma [61]. *EpCAM* overexpression promotes breast cancer invasion by activating the JNK signaling pathway and enhancing the transcriptional activity of the downstream AP-1 transcription factor [62]. Although the existing literature has not established a direct correlation between *EpCAM* and Depression (Table 1), emerging evidence suggests that *EpCAM* may contribute to depressive pathogenesis through multiple neuroinflammatory pathways. Specifically, *EpCAM* potentially promotes the secretion of pro-inflammatory cytokines IL-6 and TNF-α via ERK activation. These circulating cytokines could compromise blood–brain barrier (BBB) integrity, subsequently infiltrating the central nervous system (CNS) where they activate microglia and suppress hippocampal neurogenesis [63]. Moreover, the observed positive correlation between *EpCAM* expression and mast cell activation indicates an additional mechanism whereby histamine-mediated increases in vascular permeability may further disrupt BBB function (Figure 6C). Consequently, *EpCAM* serves as a potential molecular link underlying the comorbidity between neuropsychiatric disorders and cancers.

*GSTM2*, a Mu-class glutathione S-transferase (GST) isoenzyme, serves as a crucial regulator of cellular redox homeostasis through its catalytic activity in detoxifying ROS and electrophilic substrates, wherein functional deficiencies precipitate pathological ROS accumulation that initiates multiple pathophysiological cascades [64]. The resultant oxidative stress mediates dual activation of the MAPK signaling pathway via direct oxidation-induced conformational changes in redox-sensitive kinases (notably JNK and p38) and oxidative inactivation of MAPK phosphatases (MKPs), collectively establishing a self-amplifying cycle of sustained pathway activation that potentiates pro-inflammatory cytokine secretion [65]. Structural studies reveal that *GSTM2* exerts its regulatory function through steric hindrance of apoptosis signal-regulating kinase 1 (ASK1) oligomerization via high-affinity binding to its N-terminal tetratricopeptide repeat (TPR) domain, thereby preventing autophosphorylation and subsequent activation of the JNK/p38 signaling cascade [66]. Pathological *GSTM2* downregulation disrupts this inhibitory mechanism, leading to persistent JNK/p38 activation that transcriptionally upregulates oncogenic transcription factors (AP-1 and NF-κB), thereby establishing a pro-inflammatory milieu that induces epithelial–mesenchymal plasticity and confers metastatic competence to tumor cells [67,68,69]. Importantly, this peripherally generated cytokine storm induces BBB dysfunction through tight junction protein dysregulation, facilitating CNS infiltration of inflammatory mediators that activate microglial cells and sustain neuroinflammatory responses, while concurrently diverting tryptophan metabolism from serotonin (5-HT) biosynthesis to neurotoxic kynurenine pathway metabolites, thus mechanistically coupling *GSTM2*-mediated oxidative stress with both malignant progression and neuropsychiatric pathology [70]. Additionally, our study revealed a negative correlation between *GSTM2* expression and γδ T cell infiltration, suggesting that *GSTM2* deficiency may lead to excessive accumulation of γδ T cells in the tumor microenvironment, which is consistent with previous reports [71]. Notably, depressed patients also exhibit elevated levels of γδ T cells, implying that excessive inflammatory responses mediated by γδ T cells may represent a common pathogenic mechanism underlying both conditions [72]. However, the precise molecular mechanisms connecting γδ T cell dysregulation with disease pathogenesis remain to be fully elucidated and warrant further investigation.

Although previous studies have reported associations between *BHLHE41*/*GSTM2* and both depression and BC, this research is the first to focus on their regulatory roles through the immune system and MAPK pathway. Furthermore, the study uncovers that *EpCAM*, a gene previously linked to BC progression, may also contribute to the pathogenesis of depression, addressing a critical gap in this field. All three genes demonstrated robust diagnostic performance with an area under the ROC curve (AUC) >0.7, highlighting their potential clinical value. However, several limitations of this study should be acknowledged. First, the inherent heterogeneity between Depression and BC datasets from different databases may introduce variability due to differences in data collection protocols, sample processing methods, and population characteristics. Second, while our findings provide valuable insights into the common molecular mechanisms underlying Depression and BC, they require validation through larger, well-designed clinical studies with longitudinal follow-up to establish robust clinical correlations and therapeutic implications.

Our comprehensive analysis reveals that immune dysregulation mediated by three mechanism-related genes (*BHLHE41*, *EpCAM*, and *GSTM2*) through MAPK pathway activation, coupled with their dual promotion of neuroinflammation and EMT, forms the comorbid mechanism network between depression and BC, establishing a fundamental biological link underlying their co-occurrence.

## 4. Materials and Methods

### 4.1. Enrichment Analysis of Overlapping Genes

Depression expression profiles (20 cases, 12 controls) were obtained from the GSE76826 dataset in the GEO database [73]. BC transcriptomic data (1118 cases, 113 controls) were acquired from TCGA database [74]. To maintain analytical consistency, all samples were collected from peripheral blood mononuclear cells (PBMCs). Raw expression data were preprocessed through platform-specific annotation mapping to gene symbols and log2(X + 1) transformation using R software (version 4.4.0), generating standardized expression matrices for downstream analyses.

### 4.2. Differential Expression Analysis

Differential expression analysis was conducted using the “limma” package, version 3.60.6, with thresholds applied to Depression (|logFC| > 0.5 and *p* < 0.05) and BC (|logFC| > 1 and *p* < 0.05) datasets [75]. Differentially expressed genes (DEGs) were identified separately for each condition, with their distribution visualized through volcano plots generated in GraphPad Prism, version 8.0. The DEGs lists for Depression and BC are presented in Tables S1 and S2, respectively. Expression patterns were illustrated via hierarchical clustering heatmaps, while overlapping genes were identified using Venny 2.1 (https://bioinfogp.cnb.csic.es/, accessed on 3 March 2025).

### 4.3. Gene Ontology (GO) and Kyoto Encyclopedia of Genes and Genomes (KEGG) Pathway Analysis

Functional enrichment analysis of overlapping DEGs was conducted using the Microbiotics platform (https://www.bioinformatics.com.cn/, accessed on 3 March 2025), with significant terms identified at *p* < 0.05 [76]. Comprehensive GO annotation was performed across three categories: biological processes (BP), molecular functions (MF), and cellular components (CC). KEGG pathway analysis was subsequently employed to identify molecular pathways in Depression and BC pathogenesis.

### 4.4. Construction of the Protein–Protein Interaction (PPI) Network

A PPI network was constructed for overlapping genes using the STRING database (https://cn.string-db.org/), with a confidence score threshold of ≥0.4 to ensure biologically meaningful interactions. Isolated nodes (proteins lacking interactions) were excluded to focus on functionally connected modules. The resulting network was visualized and analyzed using Cytoscape software, version 3.10.0, with interacting nodes selected as hub genes for subsequent functional analyses.

### 4.5. Mechanism-Associated Gene Selection and Validation

LASSO regression, a machine learning algorithm employing L1 regularization to enhance model performance, was utilized for feature selection and dimensionality reduction [77]. This shrinkage-based approach effectively addresses multicollinearity while optimizing feature selection for disease classification. The diagnostic performance of candidate genes was evaluated through ROC curve analysis, with the AUC serving as a quantitative measure of discriminatory power. Genes demonstrating AUC values > 0.7 were considered clinically relevant, with higher values indicating superior predictive performance in distinguishing disease states from normal controls.

### 4.6. Immune Infiltration Analysis

To investigate immune-related mechanisms in Depression and BC progression, immune cell infiltration patterns were analyzed using the xCell algorithm, version 1.1.0 implemented in R [78]. This computational method estimates relative abundances of 64 immune cell types based on immune cell-specific gene expression signatures. Enrichment scores for each immune cell type were calculated to quantify immune microenvironment composition across samples. Spearman’s rank correlation analysis was subsequently performed to evaluate potential associations between mechanism-associated genes and immune cell infiltration patterns.

### 4.7. Single-Gene Gene Set Enrichment Analysis (GSEA) Analysis

Single-gene GSEA was conducted to investigate biological functions and molecular pathways related with mechanism-associated genes, employing comprehensive annotations from both GO and KEGG databases [79].

## Figures and Tables

**Table 1:** Literature validation of Depression and breast cancer (BC) mechanism-associated genes.

Gene	Depression	BC	References
*BHLHE41*	√	√	[32,33,34,35,36,37,38,39]
*E* *pCAM*	×	√	[40,41,42,43,44]
*GSTM2*	√	√	[45,46,47,48,49,50]

## Data Availability

The data presented in this study are openly available in GEO (https://www.ncbi.nlm.nih.gov/geo) and TCGA (https://portal.gdc.cancer.gov/) repositories.

## Supplementary Materials

The following supporting information can be downloaded at https://www.mdpi.com/article/10.3390/ijms26115229/s1.

## Abbreviations

The following abbreviations are used in this manuscript:

AUC	area under the curve
BBB	blood–brain barrier
BC	Breast cancer
CNS	central nervous system
DEGs	differentially expressed genes
EMT	epithelial–mesenchymal transition
GEO	Gene Expression Omnibus
GO	Gene Ontology
GSEA	Gene Set Enrichment Analysis
GWAS	genome-wide association studies
HPA	hypothalamic–pituitary–adrenal
IL-6	interleukin-6
KEGG	Kyoto Encyclopedia of Genes and Genomes
LASSO	least absolute shrinkage and selection operator
MAPK	mitogen-activated protein kinase
PPI	protein-protein interaction
PRS	polygenic risk scores
ROC	receiver operating characteristic
TCGA	The Cancer Genome Atlas
TNF-α	tumor necrosis factor-α
