# Supplementary figures and images for: Deciphering the Overlapping Immune Mechanism Between Depression and Breast Cancer
Source: Int J Mol Sci. 2025 May 29;26(11):5229. doi: 10.3390/ijms26115229 (PMC12155333; doi:10.3390/ijms26115229)

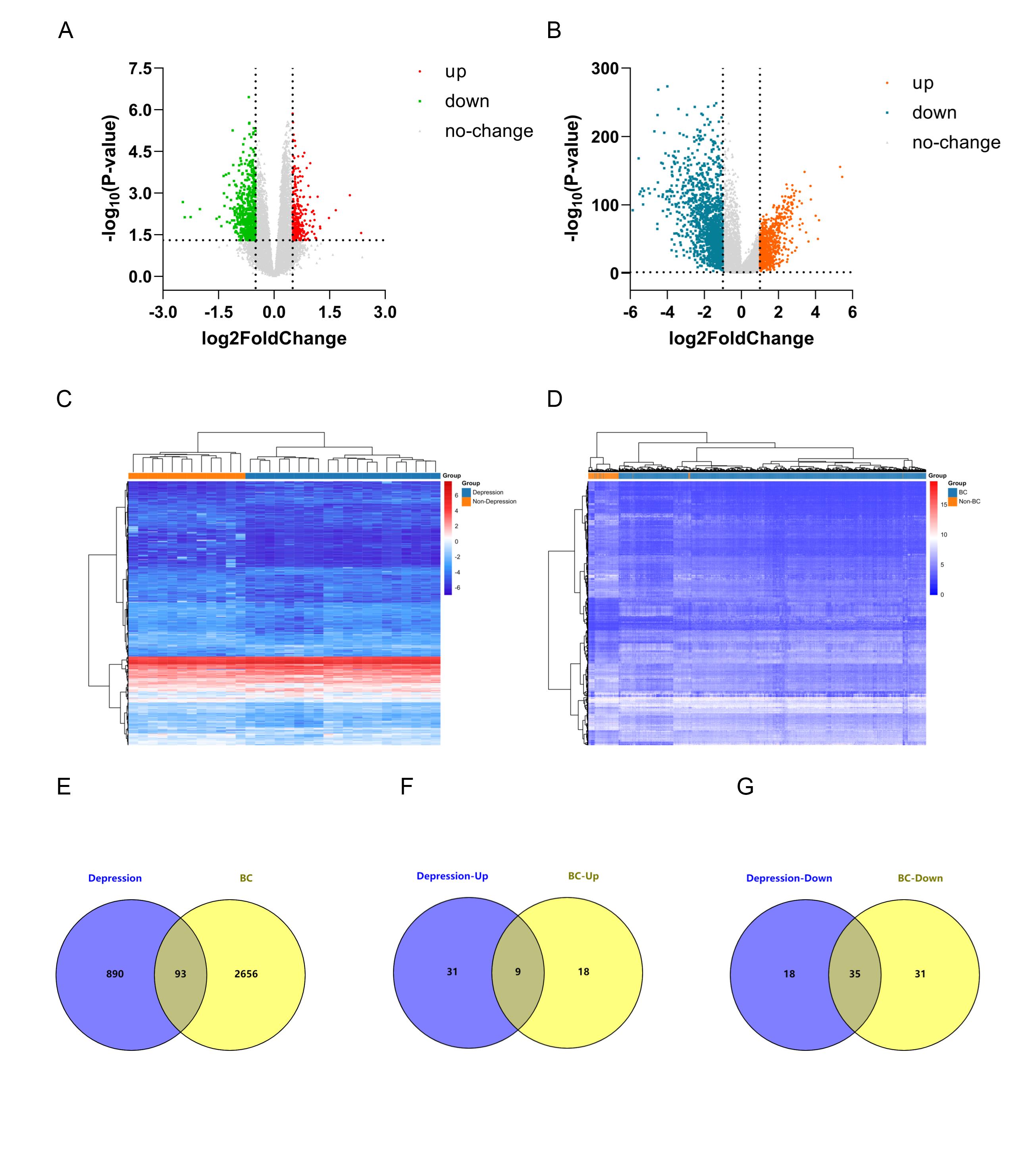

Supplement: Supplementary file 1 [file ijms-26-05229-s001.zip › Figure 1.jpg]

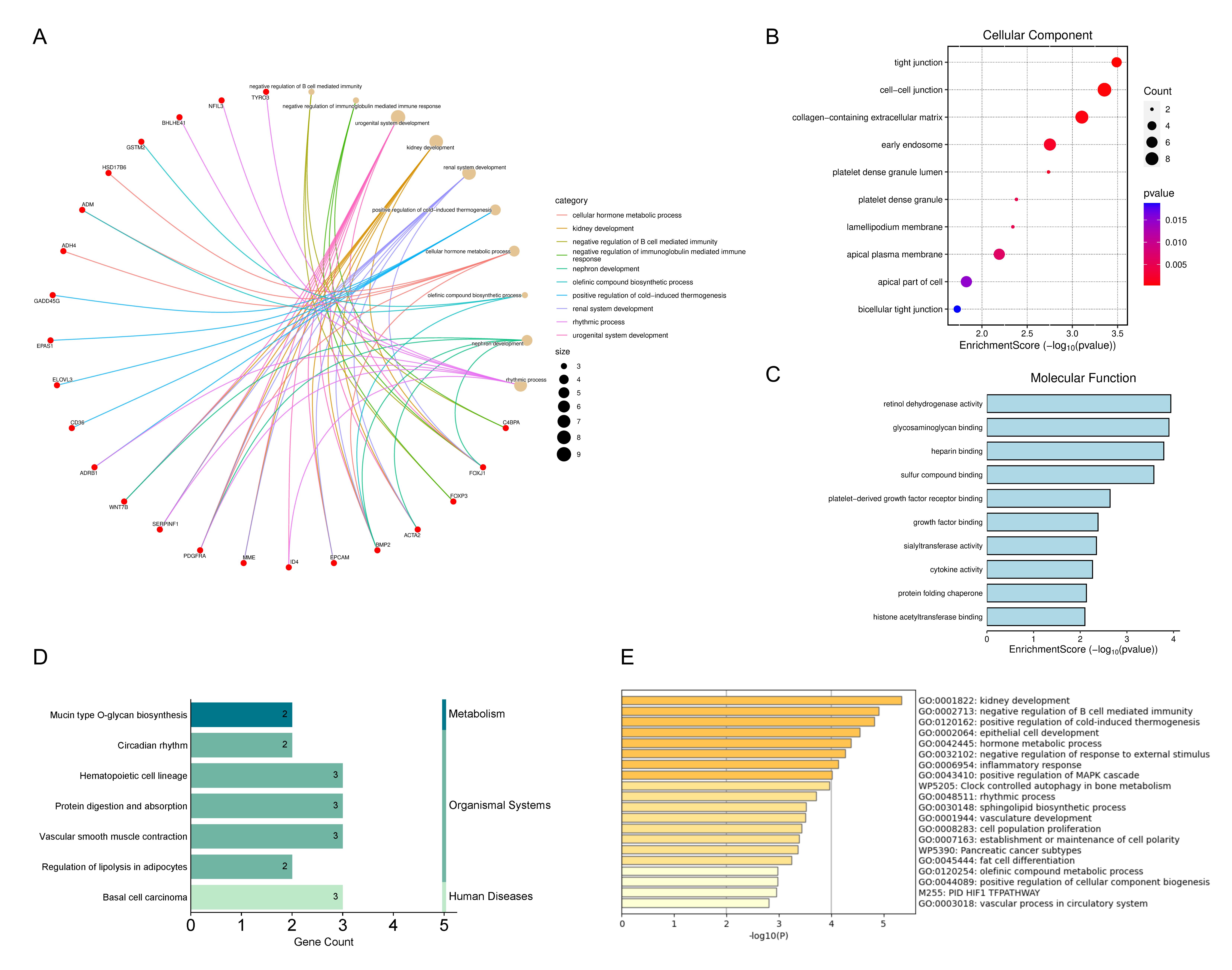

Supplement: Supplementary file 1 [file ijms-26-05229-s001.zip › Figure 2.jpg]

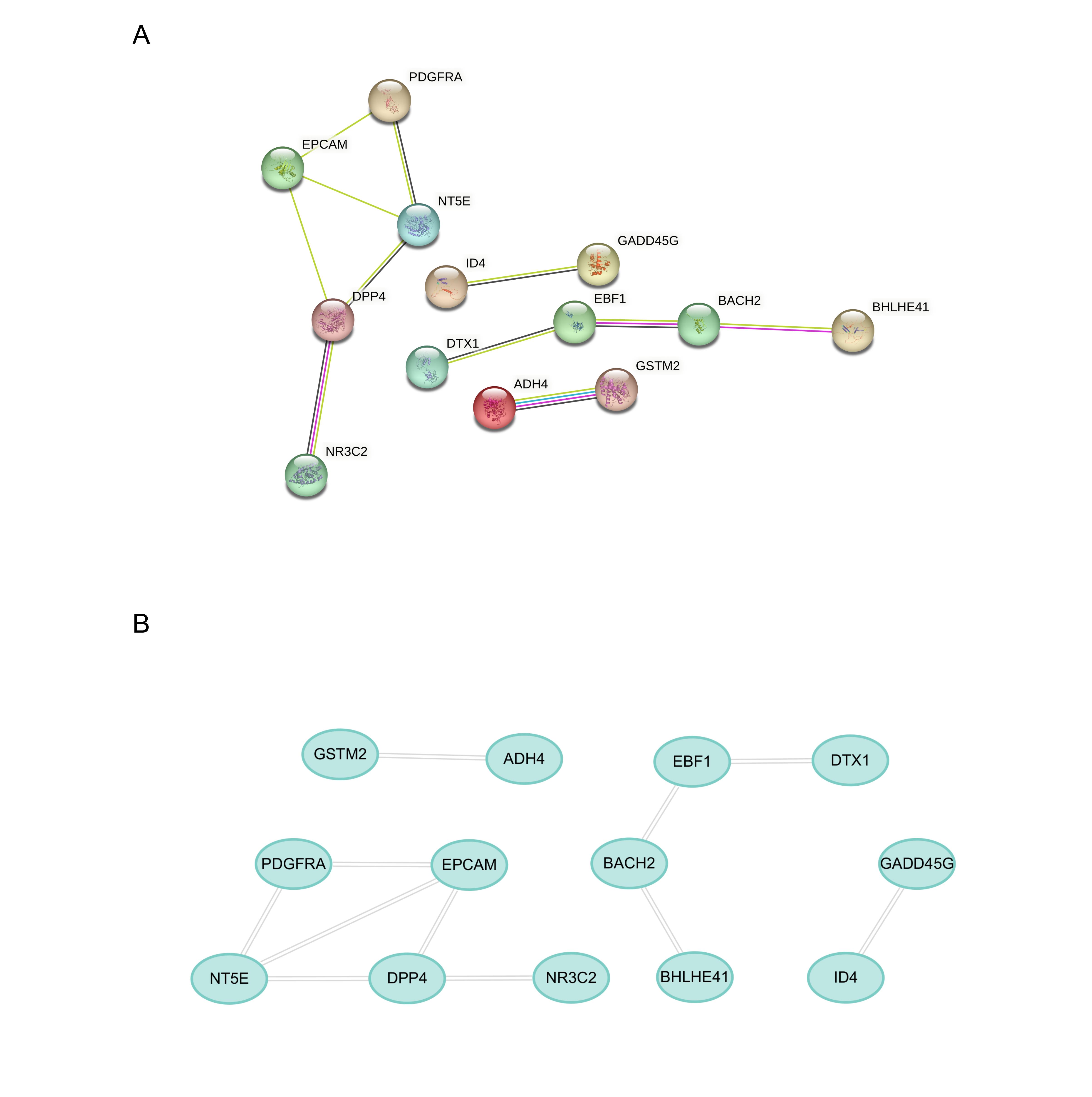

Supplement: Supplementary file 1 [file ijms-26-05229-s001.zip › Figure 3.jpg]

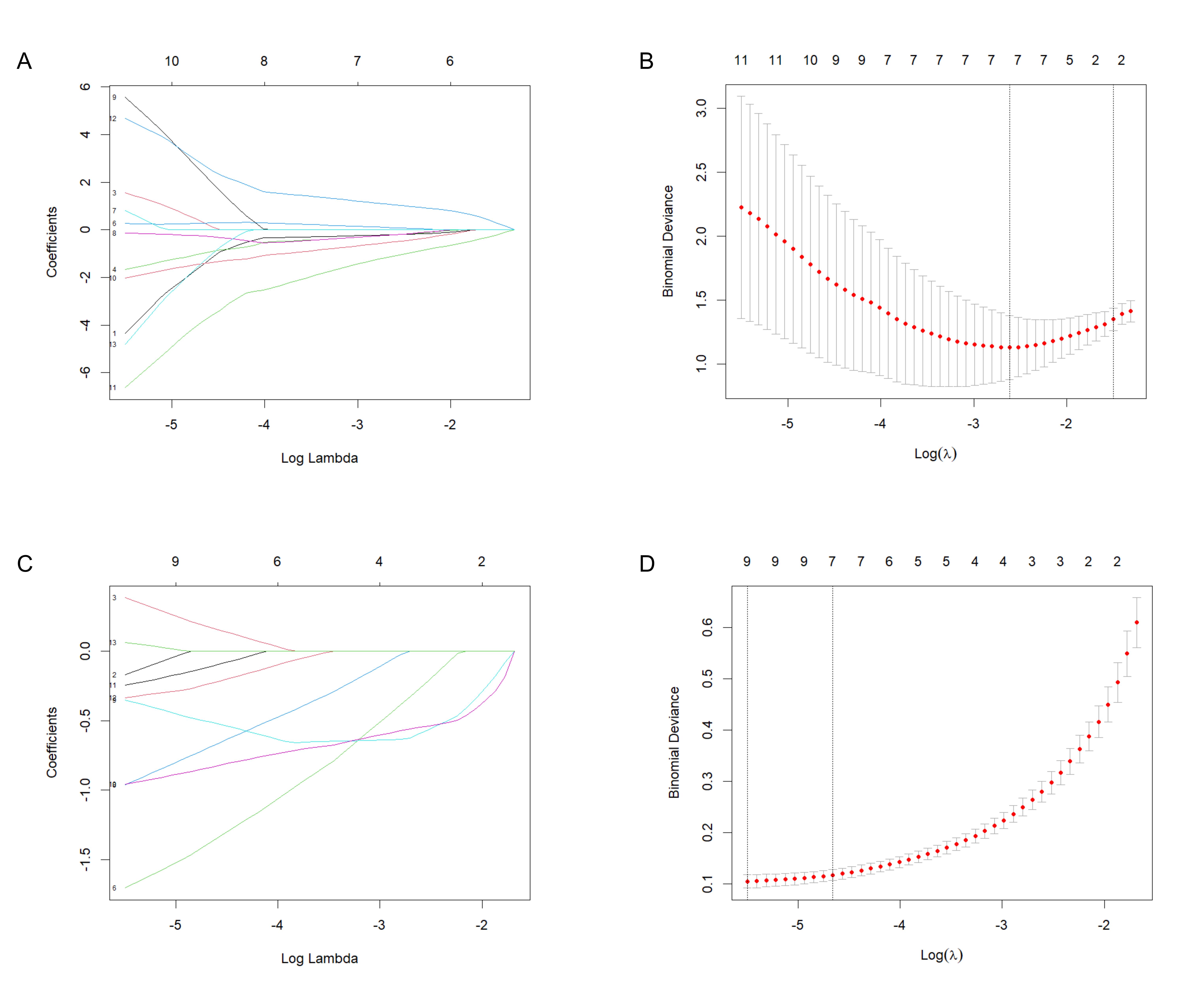

Supplement: Supplementary file 1 [file ijms-26-05229-s001.zip › Figure 4.jpg]

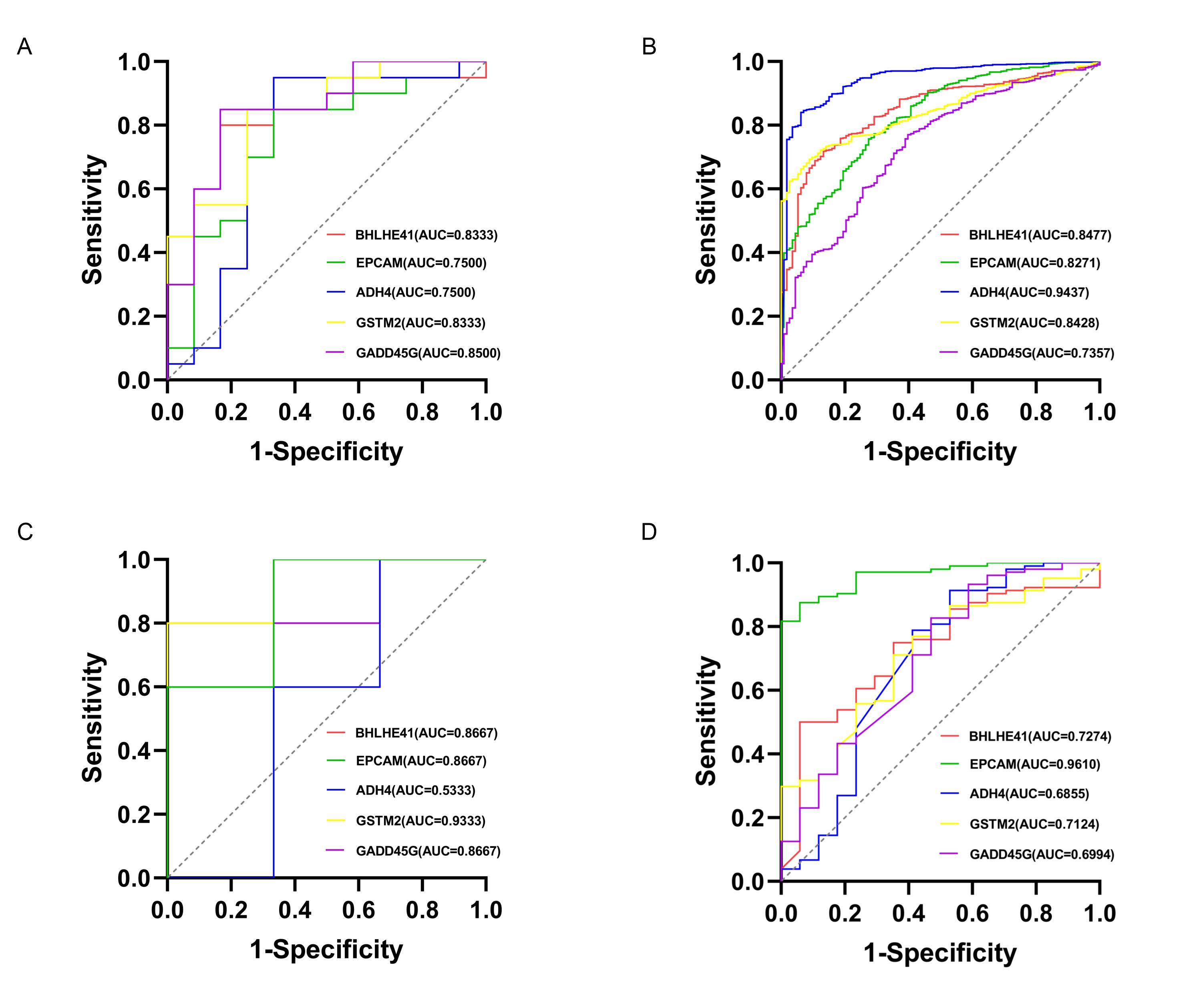

Supplement: Supplementary file 1 [file ijms-26-05229-s001.zip › Figure 5.jpg]

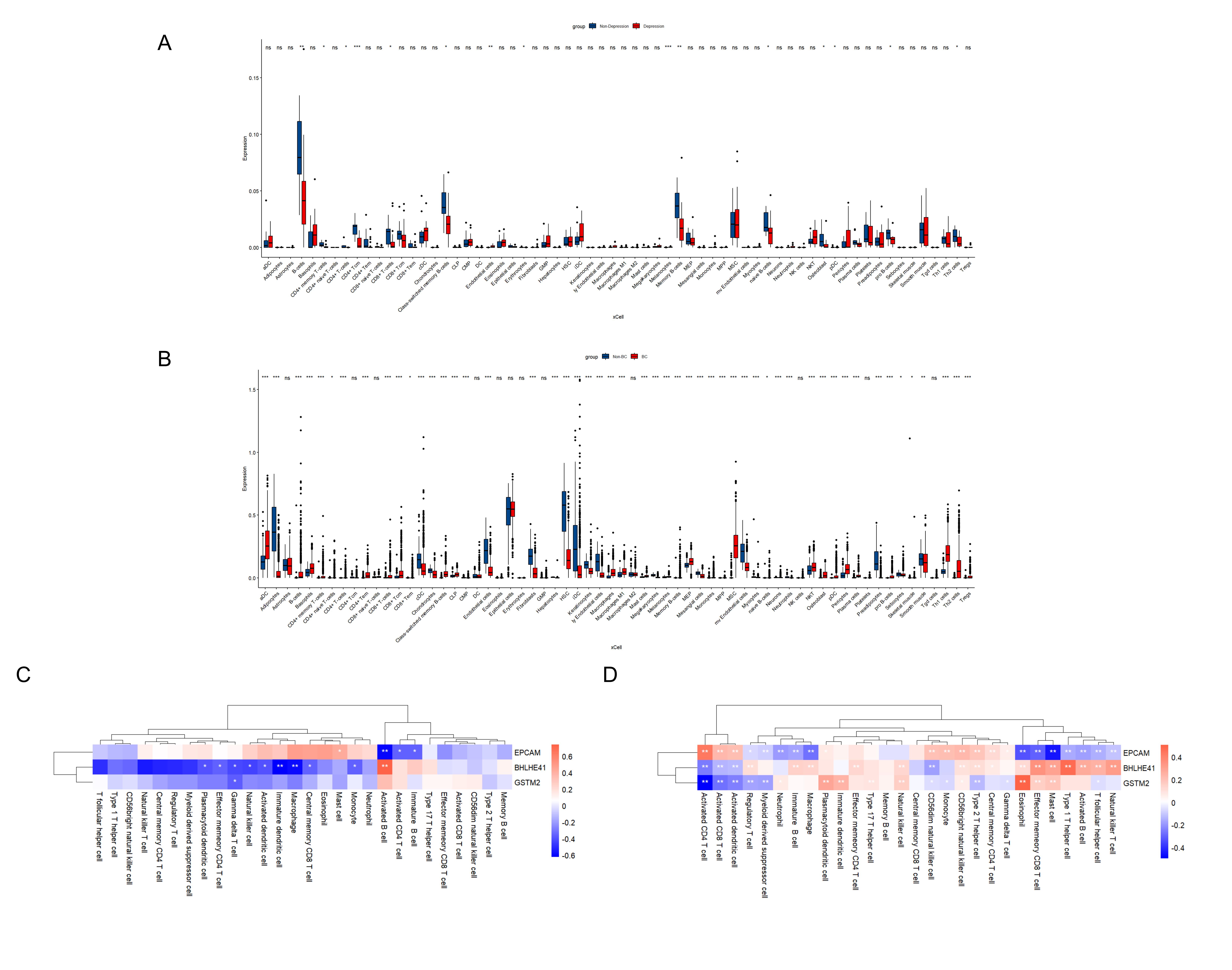

Supplement: Supplementary file 1 [file ijms-26-05229-s001.zip › Figure 6.jpg]

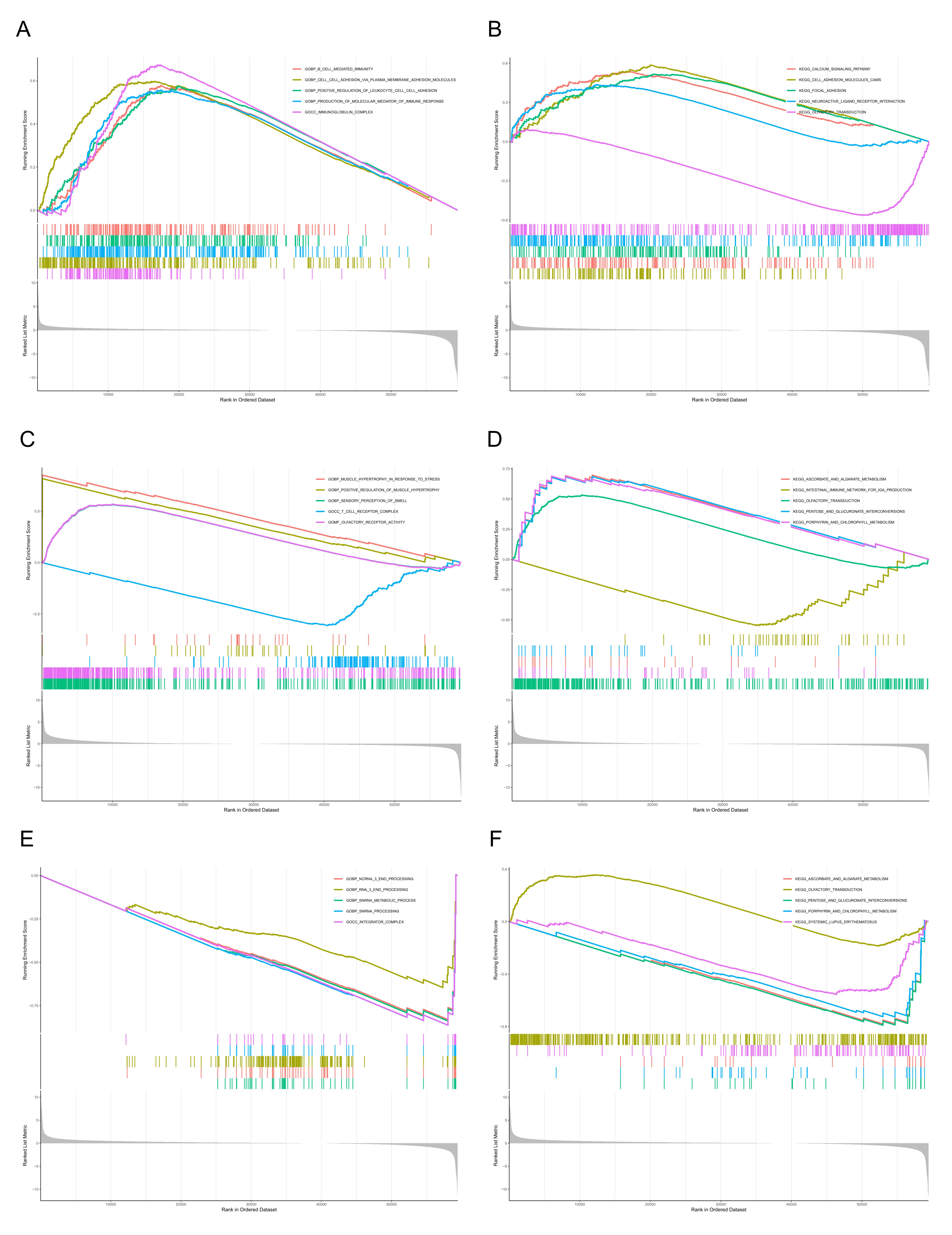

Supplement: Supplementary file 1 [file ijms-26-05229-s001.zip › Figure 7.jpg]
